# Transoral antireflux plication: a new approach for refractory GERD after laparoscopic sleeve gastroplasty

**DOI:** 10.1016/j.vgie.2025.04.002

**Published:** 2025-04-05

**Authors:** Vismaya S. Bachu, Cameron Quon, Anna H. Lee, Manar Alhanaee, Danny Issa

**Affiliations:** 1 Department of Medicine, David Geffen School of Medicine, University of California Los Angeles, Los Angeles, California, USA; 2 Vatche & Tamar Manoukian Division of Digestive Diseases, Department of Medicine, David Geffen School of Medicine, University of California Los Angeles, Los Angeles, California, USA

## Abstract

**Background and Aims:**

New-onset GERD is a common adverse event after laparoscopic sleeve gastrectomy (LSG), with limited treatment options beyond medical therapy or surgical conversion. This case study presents a minimally invasive endoscopic approach—transoral antireflux plication (TARP)—for managing post-LSG GERD.

**Methods:**

A 54-year-old woman with refractory GERD post-LSG underwent endoscopic sleeve revision and TARP. The procedure involved submucosal injection, mucosal incision, and full-thickness endoscopic suturing to reduce gastric volume and create an antireflux barrier.

**Results:**

A pH monitoring study 1 month postprocedure showed significant improvement in reflux parameters and symptom relief, independent of weight loss.

**Conclusions:**

TARP with endoscopic sleeve revision may offer a safe, minimally invasive alternative to surgery for post-LSG GERD. Further research is needed to confirm long-term efficacy and patient selection criteria.

## Background

**Figure 1:**
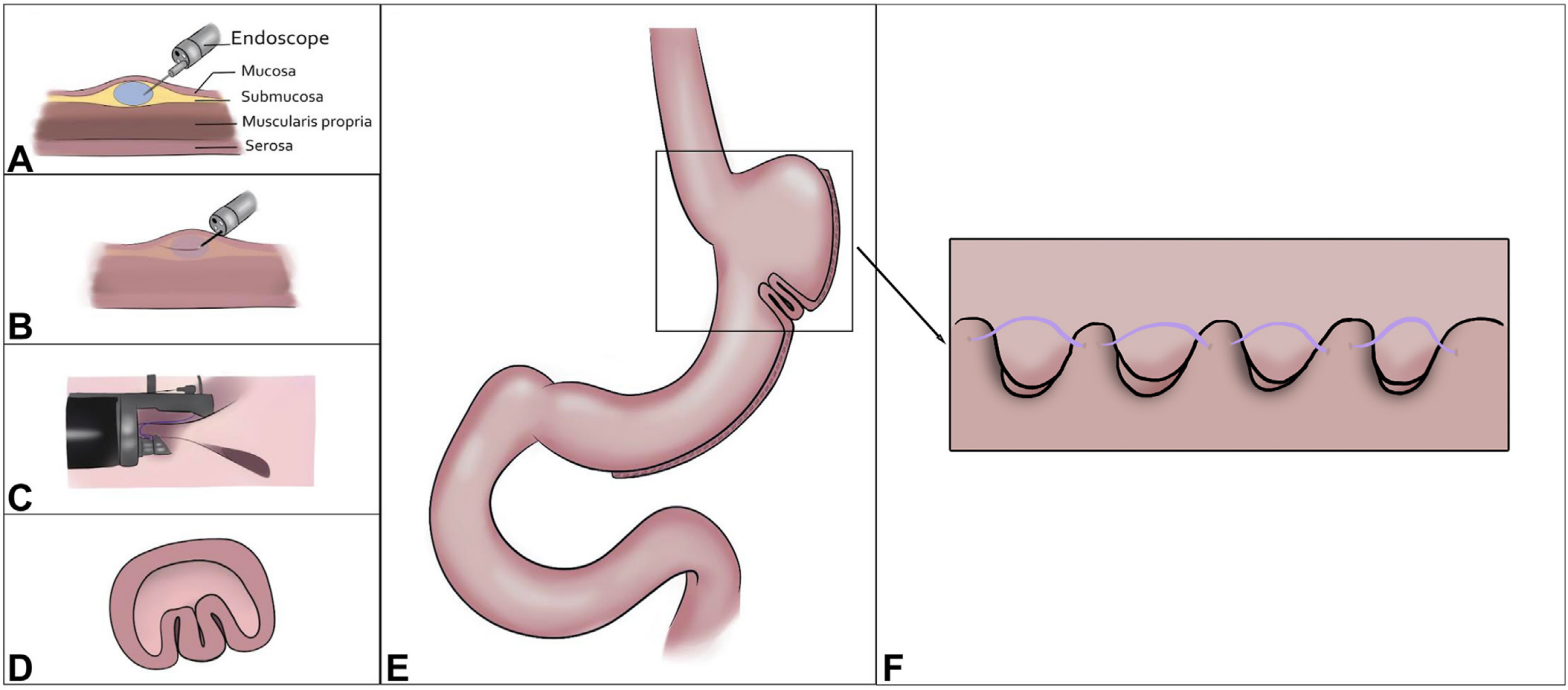
Illustration of TARP procedure. **A,** Submucosal injection, proximal to the sleeve. **B,** Incision with dissection knife. **C,** Endoscopic suturing, creating the neofundus. **D,** Cross-sectional view of the antireflux plication neofundus. **E,** Final view of TARP procedure site. **F,** Final stitch pattern. *TARP*, Transoral antireflux plication.

New-onset GERD after laparoscopic sleeve gastrectomy (LSG) is a challenging postoperative adverse event. A systematic review^1^ reported a 20% increase in reflux and an 18% prevalence of Barrett’s esophagus within 5 years after LSG. Although medical therapy is effective in mild cases, conversion to Roux-en-Y gastric bypass remains the only definitive option for refractory GERD. In this video case study, we present a novel minimally invasive approach to managing post-LSG GERD using endoscopic suturing techniques (Fig. 1).

## Case presentation

A 54-year-old woman with type 2 diabetes, hyperlipidemia, and a history of remote LSG for morbid obesity developed new-onset GERD 1 year postoperatively. Although she initially achieved significant weight loss, she regained 50 pounds after her procedure over 2 years, with her body mass index reaching 40 kg/m^2^. Her GERD symptoms persisted despite optimized proton pump inhibitor (PPI) therapy, dietary, and lifestyle modifications. Upper endoscopy revealed erosive esophagitis, a dilated gastric pouch, and abnormal pH monitoring confirming persistent GERD despite PPI therapy. The patient underwent an endoscopic revision of the gastric sleeve, combined with a transoral antireflux plication procedure for her reflux disease.

## Endoscopic methods

**Figure 2:**
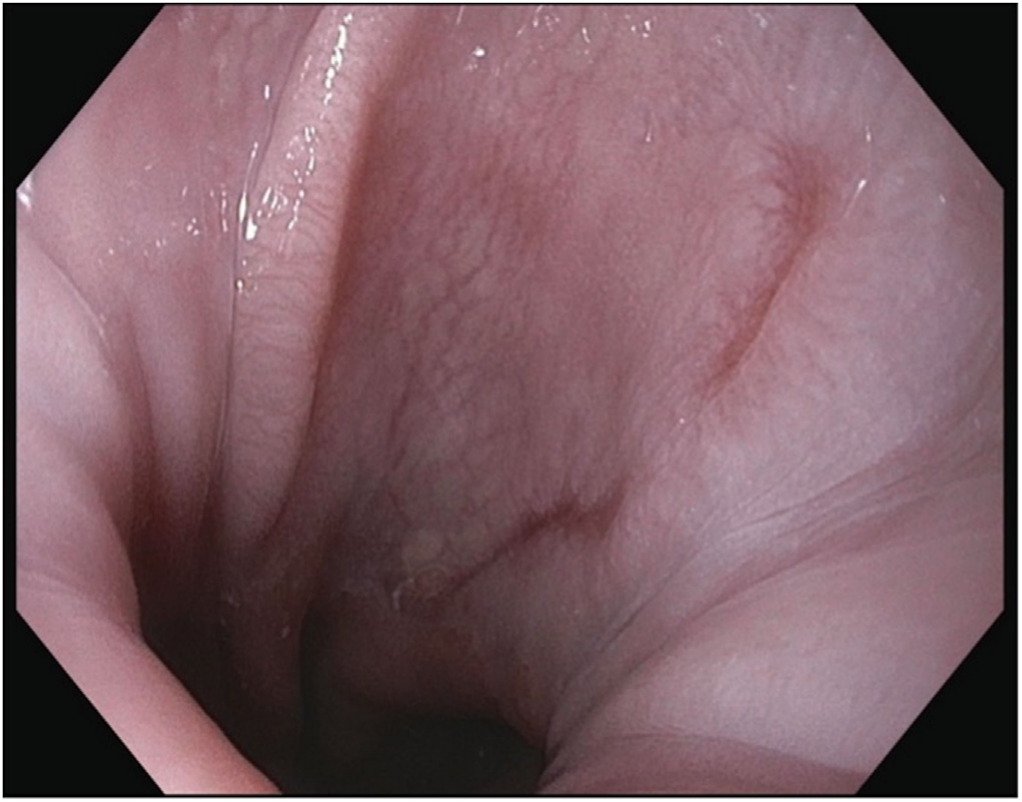
Initial endoscopic evaluation demonstrated evidence of previous sleeve gastrectomy with a grade A erosive esophagitis.

**Figure 3:**
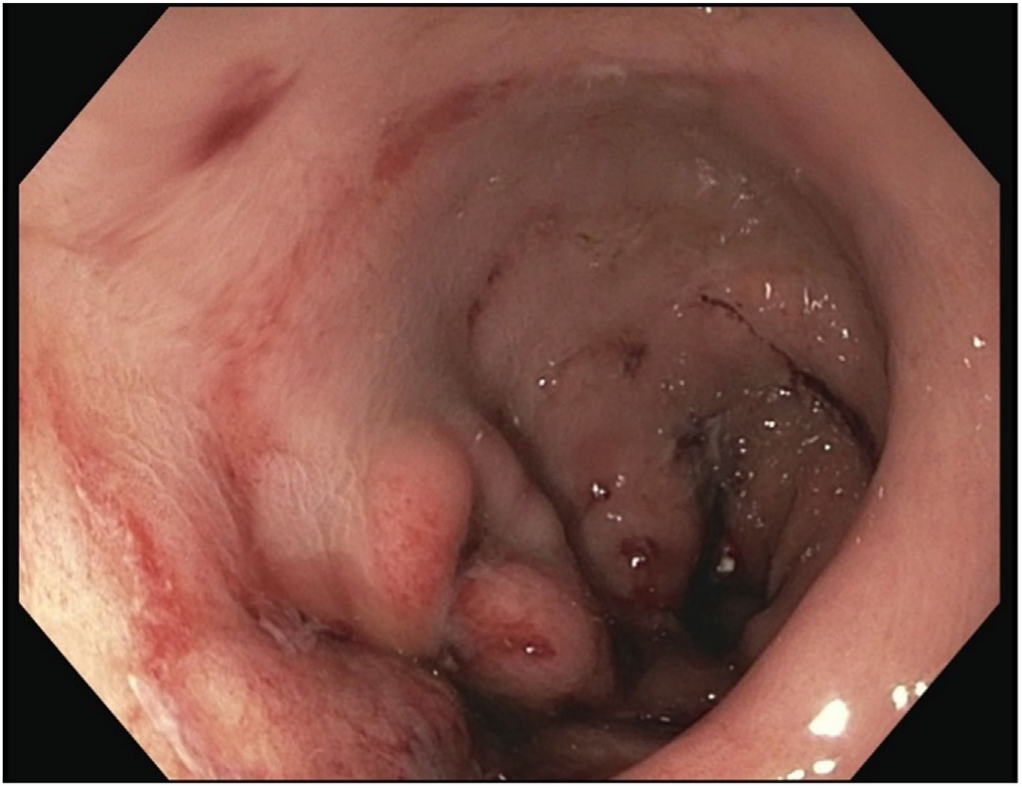
Endoscopic view of postrevision gastric sleeve.

Initial endoscopic evaluation demonstrated evidence of previous sleeve gastrectomy with significant enlargement of the gastric lumen and Grade A erosive esophagitis (Fig. 2). The gastric sleeve was revised endoscopically per re-sleeve protocol (Fig. 3). Four sutures were placed in U-pattern, with an average of 7 bites per suture, to achieve the desired reduction in gastric volume. Subsequently, an antireflux barrier was created using the steps that follow.

### Step 1: submucosal injection

Saline mixed with methylene blue was injected at approximately 4 cm distal to the gastroesophageal junction and proximal to the sleeve revision. The 4-cm mark was chosen to create a fundus space.

### Step 2: mucosal incision

A semicircumferential transverse mucosal incision, approximately 6 cm in length, was made using an I-shaped endoscopic submucosal dissection knife with (Endocut Q [Erbe Elektromedizin GmbH, Tübingen, Germany], effect 3, cut duration 2). The goal was to achieve a clear exposure of the muscle layer.

### Step 3: endoscopic suturing

**Figure 4:**
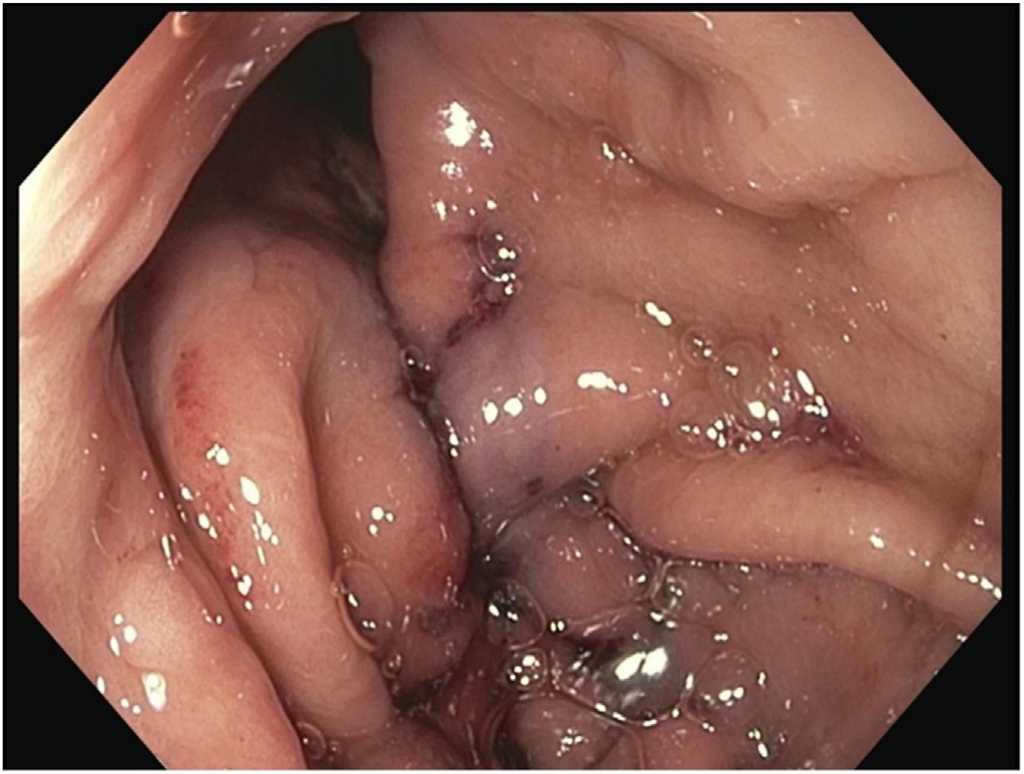
View of antireflux plication procedure immediately after the intervention.

**Figure 5:**
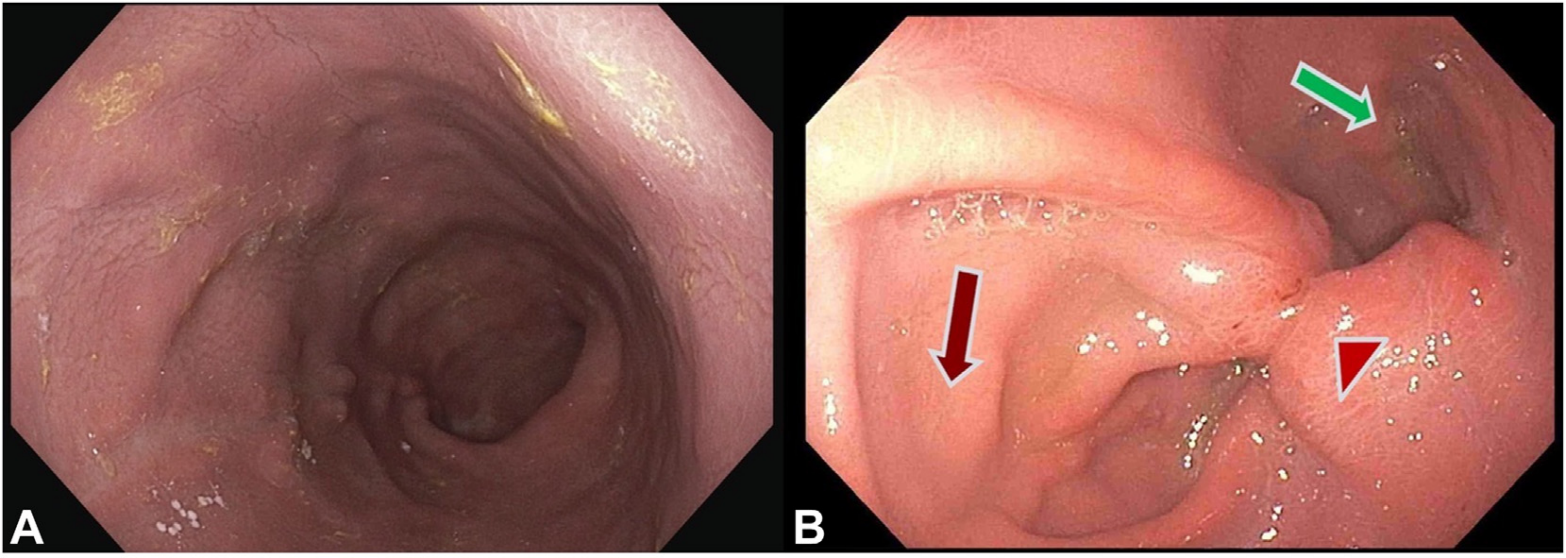
Endoscopic view of a dilated sleeve before (**A**) and 4 weeks after transoral antireflux plication procedure (**B**), demonstrating narrowed lumen (*green arrow*), antireflux barrier (*red arrowhead*), and neofundus (*red arrow*).

A dual-channel endoscope with a suturing device was used for full-thickness sutures. A helix device ensured the inclusion of the muscularis layer, and 1 running suture was placed along the incision line. A second reinforcing suture was added for prolongation of the plication, and a cinch was applied to tighten and cut the sutures (Fig. 4). The end result was the creation of an antireflux barrier and a neofundus to minimize acid reflux (Fig. 5).

Post-transoral antireflux plication pH monitoring performed in the early phase intentionally (1 month after the intervention) revealed marked improvement in GERD parameters and an adequate response to PPI (Table 1).

**Table 1:** pH parameters on day 1 and day 2 of PPI before and after TARP procedure

	Before TARP		After TARP (4 weeks later)	
	pH study day 1	pH study day 2	pH study day 1	pH study day 2
No. of refluxes				
Total (ref <5.3)	29	27	29	4∗
Upright (ref <6.9)	23	25	9	4∗
Supine (ref <6.7)	7	3∗	20	0∗
% time pH <4.0				
Total (ref <5.3)	5.2∗	10.2	5.2∗	0.6∗
Upright (ref <6.9)	1.5∗	10.6	1.5∗	0.6∗
Supine (ref <6.7)	13.8	4.9∗	13.8	0∗
DeMeester score (ref <14.72)	28.4	32.1	18.7	1.9∗

*PPI*, Proton pump inhibitor; *ref*, reference range; *TARP*, transoral antireflux plication.

∗ Within normal ranges.

## Conclusions

This case highlights the potential of a transoral antireflux plication as a minimally invasive option for treating GERD after LSG. The procedure effectively reduces the gastric pouch size, creates an antireflux barrier, and preserves a neofundus to minimize acid reflux. Early postintervention pH studies demonstrated significant symptom improvement, independent of weight loss. Further research is needed to optimize patient selection and validate long-term outcomes of this technique for post-LSG GERD.

## Patient Consent

The patient in this article has given written informed consent to publication of the case details.

## Disclosures

D. Issa has served as a consultant for Boston Scientific and as a speaker for Eli Lilly. All other authors disclosed no financial relationships.

## Supplementary data

Video 1 Endoscopic management of post-LSG GERD using a combined approach of endoscopic sleeve revision and transoral antireflux plication (TARP). The procedure includes submucosal injection, mucosal incision, and full-thickness suturing to reduce gastric volume and create a neofundus, functionally restoring an antireflux barrier. Key time stamps are noted below: 00:27 – Background 00:53 – Case presentation 00:26 – Initial endoscopic examination 00:39 – Endoscopic sleeve revision 02:03 – TARP procedure 03:26 – View of antireflux plication 04:22 – Postprocedural pH study results 04:48 – Conclusions
